# In-person nursing education as an ethical and life imperative: ABEn’s achievement in defending quality training

**DOI:** 10.1590/0034-7167.2025780402

**Published:** 2025-08-08

**Authors:** Jacinta de Fatima Sena da Silva, Célia Alves Rozendo

**Affiliations:** I Fiocruz, Brasília. Professor of the Graduate Program in Public Health Policies, Angicos-Núcleo de Educação Popular em Saúde e Programa de Saúde, Ambiente e Trabalho. Universidade de Brasília, Department of Public Health. Researcher and Professor. President of the Brazilian Nursing Association (2022-2025 term)). Brasília, Federal District, Brazil; II Professor Emeritus, Universidade Federal de Alagoas. Director of Education of the Brazilian Nursing Association (2022-2025 term). Maceió, Alagoas, Brazil

During the 86^th^ Brazilian Nursing Week, the Brazilian Nursing Association (In Portuguese, *Associação Brasileira de Enfermagem -* ABEn), aware of its social responsibility and its commitment to the struggles of the category and the population, celebrates the achievement of guaranteeing in-person teaching in nursing, established through Decree 12,456 of May 19, 2025^(1)^, which provides for the provision of distance learning (DL) courses by higher education institutions. This achievement is the result of many confrontations, decades-long historical struggles, old and recent mobilizations led by ABEn as well as political articulation and collective work in defense of quality training in nursing.

The in-person nature of teaching, which we advocate for all health courses, allows students to develop technical and interpersonal skills that are essential for professional practice, ensuring that future workers are prepared to act in response to individuals’ and groups’ health needs in the Brazilian Health System (In Portuguese, *Sistema Único de Saúde* - SUS) and collaboratively, in interprofessional teams.

The ban on offering undergraduate nursing courses through DL interrupts the alarming scenario of the unbridled and disorderly offering of courses in this modality. According to data from the 2023 Census of Higher Education in Brazil^(2)^, the number of DL courses increased by 232% in the last five years. The places offered, in turn, corresponded to 77.7% of the total places in higher education, indicating a growth of 167.5%, while the offer of places in in-person courses suffered a reduction of 13.5% in the period.

This worrying scenario regarding the supply of courses and vacancies is accentuated by the assessment of courses. The Summary Report of the Area: Nursing^(3)^ of the last Brazilian National Student Performance Exam, carried out in 2023 and released in 2025, presented serious data regarding student performance, the results of which indicated that all courses offered in the DL modality that participated in the exam obtained grades 1 and 2. In the in-person teaching modality, the scenario is less drastic, but still challenging. The data express a critical reality of nursing training in Brazil, characterized by disorderly expansion of the offer of courses and vacancies, weak regulation and insufficient assessment, which implies rethinking the entire regulatory and assessment process.

ABEn’s position on in-person teaching in nursing is reflected in the new Brazilian National Curricular Guidelines for the Undergraduate Course in Nursing (In Portuguese, *Diretrizes Curriculares Nacionais do Curso de Graduação em Enfermagem* - DCN/Enf), approved by the Brazilian National Education Council in July 2024, whose opinion^(4)^ has been published but has not yet been approved by the Ministry of Education. The DCN/Enf in the approval process emphasize nursing’s practical and relational nature, and reinforce the training of nurses beyond the acquisition of theoretical and practical knowledge.

Professional nursing practice involves direct contact with users, requiring empathy, assertive communication and rapid decision-making in critical situations, which demands a broad set of knowledge and skills (ethical, technical, political, attitudinal) that are only established in in-person experiences, experiences and interactions achieved through supervised practical activities, which are difficult to replicate in virtual environments. In this regard, in-person teaching constitutes an ethical and essential life imperative to guarantee the quality of care and health care for the population.

Furthermore, it is essential to highlight that the SUS sustainability depends on the qualification of its workers, and the guarantee of effective and welcoming care requires in-person health training. Only in this way is it possible to guarantee workers who are able to meet the population’s growing and complex health demands, and ensure qualified care from the perspective of health as a right of citizenship.

The achievement expressed by Decree 12,456 of May 19, 2025 represents progress that deserves to be celebrated, demonstrating collective strength, fighting capacity and the power of articulation. However, it also entails the reaffirmation of commitments, vigilance and permanent mobilization for the struggles that remain on the agenda in defense of quality education and training for nursing workers, based on ethics in defense of life on the planet, solidarity among peoples and social justice.
