# Supplementary material for: In-person nursing education as an ethical and life imperative: ABEn’s achievement in defending quality training
Source: Rev Bras Enferm. 2025 Aug 8;78(4):e780402. doi: 10.1590/0034-7167.2025780402 (PMC12334132; doi:10.1590/0034-7167.2025780402)
Supplement: Translation

# Translation: Presencialidade do ensino na enfermagem como imperativo ético e de vida: conquista da ABEn na defesa da formação de qualidade

**DOI:** 10.1590/0034-7167.2025780402pt

**Authors:** Jacinta de Fatima Sena da Silva, Célia Alves Rozendo

**Affiliations:** I Fiocruz, Brasília. Docente de Programa de Pós-Graduação em Políticas Públicas de Saúde, Angicos-Núcleo de Educação Popular em Saúde e Programa de Saúde, Ambiente e Trabalho. Universidade de Brasília, Departamento de Saúde Coletiva. Pesquisadora e Docente. Presidenta da Associação Brasileira de Enfermagem (Gestão 2022-2025). Brasília, Distrito Federal, Brasil; II Professora Titular Emérita da Universidade Federal de Alagoas. Diretora de Educação da Associação Brasileira de Enfermagem (Gestão 2022-2025). Maceió, Alagoas, Brasil

No decurso da 86^a^ Semana Brasileira de Enfermagem, a Associação Brasileira de Enfermagem (ABEn), consciente de sua responsabilidade social e de seu compromisso com as lutas da categoria e da população, celebra a conquista da garantia do ensino presencial na enfermagem, estabelecida por meio do Decreto n^o^ 12.456, de 19 de maio de 2025^(1)^, que dispõe sobre a oferta de cursos de educação a distância (EaD) pelas instituições de ensino superior. Tal conquista é fruto de muitos enfrentamentos, lutas históricas de décadas, mobilizações antigas e recentes lideradas pela ABEn, assim como articulação política e trabalho coletivo em defesa da formação de qualidade na enfermagem.

A presencialidade do ensino, a qual defendemos para todos os cursos da área da saúde, permite que estudantes desenvolvam habilidades técnicas e interpessoais indispensáveis ao exercício profissional, assegurando que futuras(os) trabalhadoras(es) estejam preparadas(os) para atuar mediante as necessidades de saúde das pessoas e grupos no Sistema Único de Saúde (SUS) e de forma colaborativa, em equipes interprofissionais.

A proibição da oferta de cursos de graduação em enfermagem EaD interrompe o cenário alarmante da oferta desenfreada e desordenada de cursos nessa modalidade. Segundo os dados do Censo 2023 da Educação Superior no Brasil^(2)^, o número de cursos EaD teve acréscimo de 232% nos últimos cinco anos. As vagas ofertadas, por sua vez, corresponderam a 77,7% do total de vagas no ensino superior, apontando um crescimento de 167,5%, enquanto a oferta de vagas em cursos presenciais sofreu redução de 13,5% no período.

Este cenário preocupante da oferta de cursos e vagas é acentuado pela avaliação dos cursos. O Relatório Síntese de Área: Enfermagem^(3)^ do último Exame Nacional de Desempenho dos Estudantes, realizado em 2023 e divulgado em 2025, apresentou dados graves em relação ao desempenho dos estudantes, cujos resultados apontaram que todos os cursos ofertados na modalidade EaD que participaram do exame obtiveram conceitos 1 e 2. Na modalidade ensino presencial, o cenário é menos drástico, mas ainda desafiador. Os dados expressam uma realidade crítica da formação de enfermeira(os) no Brasil, caracterizada por expansão desordenada da oferta de cursos e vagas, regulação frágil e avaliação insuficiente, o que implica repensar todo o processo regulatório e avaliativo.

O posicionamento da ABEn sobre o ensino presencial na enfermagem está refletido nas novas Diretrizes Curriculares Nacionais do Curso de Graduação em Enfermagem (DCN/Enf), aprovadas pelo Conselho Nacional de Educação em julho de 2024, cujo parecer^(4)^ encontra-se publicado, mas ainda não homologado pelo Ministério da Educação. As DCN/Enf em processo de homologação enfatizam a natureza prática e relacional da enfermagem, e reforçam a formação de enfermeiras(os) para além da aquisição de conhecimentos teóricos e práticos.

O exercício profissional na enfermagem envolve contato direto com usuários, exigindo empatia, comunicação assertiva e tomada de decisão rápida em situações críticas, o que demanda um conjunto ampliado de saberes e habilidades (éticos, técnicos, políticos, atitudinais) que apenas se firmam nas experiências, vivências e interações presenciais alcançadas por meio de atividades práticas supervisionadas, difíceis de serem replicadas em ambientes virtuais. Nesse sentido, a presencialidade do ensino constitui um imperativo ético e de vida essencial para garantir a qualidade do cuidado e da atenção à saúde da população.

Além disso, é fundamental destacar que a sustentabilidade do SUS depende da qualificação de suas(seus) trabalhadoras(es), e a garantia de atendimento resolutivo e acolhedor requer a formação presencial na área da saúde. Somente assim é possível garantir trabalhadoras(es) aptas(os) a atender às crescentes e complexas demandas de saúde da população, e assegurar cuidado qualificado e na perspectiva da saúde como direito de cidadania.

A conquista expressa pelo Decreto n^o^ 12.456, de 19 de maio de 2025, significa um avanço que merece comemoração, demonstrando força coletiva, capacidade de luta e poder de articulação. Porém, acarreta também a reafirmação de compromissos, a vigilância e a mobilização permanente para as lutas que seguem na pauta em defesa do ensino e da formação de qualidade de trabalhadores de enfermagem, fundamentadas na ética em defesa das vidas no planeta, na solidariedade entre os povos e na justiça social.

# Translation: La formación presencial de enfermería como imperativo ético y de vida: el logro de la ABEn en la defensa de una formación de calidad

**DOI:** 10.1590/0034-7167.2025780402es

**Authors:** Jacinta de Fatima Sena da Silva, Célia Alves Rozendo

**Affiliations:** I Fiocruz, Brasília. Profesor del Programa de Postgrado en Políticas de Salud Pública, Angicos-Núcleo de Educação Popular em Saúde e Programa de Saúde, Ambiente e Trabalho. Universidade de Brasília, Departamento de Salud Pública. Investigadora y profesora. Presidenta de la Asociación Brasileña de Enfermería (período 2022-2025). Brasília, Distrito Federal, Brasil; II Profesor Emérito de la Universidade Federal de Alagoas. Director de Educación de la Asociación Brasileña de Enfermería (período 2022-2025). Maceió, Alagoas, Brasil

Durante la 86ª Semana Brasileña de Enfermería, la Asociación Brasileña de Enfermería (En portugués, *Associação Brasileira de Enfermagem* - ABEn), consciente de su responsabilidad social y su compromiso con las luchas de la categoría y de la población, celebra el logro de garantizar la enseñanza presencial en enfermería, establecido a través del Decreto 12.456, del 19 de mayo de 2025^(1)^, que prevé la impartición de cursos de educación a distancia (EaD) por parte de instituciones de educación superior. Este logro es el resultado de múltiples confrontaciones, luchas históricas de décadas, movilizaciones antiguas y recientes lideradas por la ABEn, así como de la articulación política y el trabajo colectivo en defensa de la formación de calidad en enfermería.

El carácter presencial de la enseñanza, que preconizamos para todos los cursos del área de la salud, permite que los estudiantes desarrollen habilidades técnicas e interpersonales esenciales para el ejercicio profesional, garantizando que los futuros trabajadores estén preparados para actuar en respuesta a las necesidades de salud de los individuos y grupos en el Sistema Único de Salud (En portugués, *Sistema Único de Saúde* - SUS) y de forma colaborativa, en equipos interprofesionales.

La prohibición de ofrecer cursos de enfermería de pregrado a distancia interrumpe el alarmante panorama de la oferta descontrolada y desordenada de cursos en esta modalidad. Según datos del Censo de 2023 de Educación Superior en Brasil^(2)^, el número de EaD aumentó un 232 % en los últimos cinco años. Las plazas ofertadas, a su vez, representaron el 77,7 % del total de plazas en educación superior, lo que indica un crecimiento del 167,5 %, mientras que la oferta de plazas en cursos presenciales sufrió una reducción del 13,5 % en el mismo período.

Este preocupante panorama en cuanto a la oferta de cursos y plazas se ve acentuado por la evaluación de los cursos. El Informe Resumido del Área de Enfermería^(3)^ del último Examen Nacional de Desempeño Estudiantil, realizado en 2023 y publicado en 2025, presentó datos relevantes sobre el desempeño estudiantil. Los resultados indicaron que todos los cursos ofrecidos en la modalidad EaD que participaron en el examen obtuvieron calificaciones de 1 y 2. En la modalidad presencial, el panorama es menos drástico, pero sigue siendo desafiante. Los datos reflejan una realidad crítica de la formación en enfermería en Brasil, caracterizada por una expansión desordenada de la oferta de cursos y plazas, una regulación deficiente y una evaluación insuficiente, lo que implica replantear todo el proceso regulatorio y de evaluación.

La postura de la ABEn sobre la docencia presencial en enfermería se refleja en las nuevas Directrices Curriculares Nacionales para el Grado en Enfermería (En portugués, *Diretrizes Curriculares Nacionais do Curso de Graduação em Enfermagem* - DCN/Enf), aprobadas por el Consejo Nacional de Educación en julio de 2024, cuyo dictamen^(4)^ ya se publicó, pero aún no ha sido aprobado por el Ministerio de Educación. Las DCN/Enf, en proceso de aprobación, enfatizan el carácter práctico y relacional de la enfermería y refuerzan la formación de las enfermeras más allá de la adquisición de conocimientos teóricos y prácticos.

La práctica profesional de enfermería implica el contacto directo con los usuarios, lo que requiere empatía, comunicación asertiva y una rápida toma de decisiones en situaciones críticas. Esto exige un amplio conjunto de conocimientos y habilidades (éticas, técnicas, políticas y actitudinales) que solo se adquieren en experiencias presenciales, experiencias e interacciones logradas mediante actividades prácticas supervisadas, y que son difíciles de replicar en entornos virtuales. En este sentido, la docencia presencial constituye un imperativo ético y esencial para garantizar la calidad de la atención y la salud de la población.

Además, es fundamental destacar que la sostenibilidad del SUS depende de la cualificación de sus trabajadores, y la garantía de una atención eficaz y acogedora requiere formación presencial en el área de la salud. Solo así es posible garantizar trabajadores capaces de atender las crecientes y complejas demandas de salud de la población, y asegurar una atención cualificada desde la perspectiva de la salud como derecho de ciudadanía.

El logro expresado por el Decreto 12.456 del 19 de mayo de 2025 representa un avance digno de ser celebrado, demostrando fuerza colectiva, capacidad de lucha y capacidad de articulación. Sin embargo, también implica la reafirmación de los compromisos, la vigilancia y la movilización permanente por las luchas que siguen en agenda en defensa de la educación y la formación de calidad para el personal de enfermería, basadas en la ética en defensa de la vida en el planeta, la solidaridad entre los pueblos y la justicia social.
